# Automated Screening for Abdominal Aortic Aneurysm in CT Scans under Clinical Conditions Using Deep Learning

**DOI:** 10.3390/diagnostics11112131

**Published:** 2021-11-17

**Authors:** Alena-K. Golla, Christian Tönnes, Tom Russ, Dominik F. Bauer, Matthias F. Froelich, Steffen J. Diehl, Stefan O. Schoenberg, Michael Keese, Lothar R. Schad, Frank G. Zöllner, Johann S. Rink

**Affiliations:** 1 Computer Assisted Clinical Medicine, Mannheim Institute for Intelligent Systems in Medicine, Medical Faculty Mannheim, Heidelberg University, Theodor-Kutzer-Ufer 1-3, D-68167 Mannheim, Germany; Alena-Kathrin.Golla@medma.uni-heidelberg.de (A.-K.G.); Christian.Toennes@medma.uni-heidelberg.de (C.T.); Tom.Russ@medma.uni-heidelberg.de (T.R.); Dominik.Bauer@medma.uni-heidelberg.de (D.F.B.); Lothar.Schad@medma.uni-heidelberg.de (L.R.S.); Frank.Zoellner@medma.uni-heidelberg.de (F.G.Z.); 2 Department of Radiology and Nuclear Medicine, University Medical Center Mannheim, Theodor-Kutzer-Ufer 1-3, D-68167 Mannheim, Germany; Matthias.Froelich@medma.uni-heidelberg.de (M.F.F.); steffen.diehl@medma.uni-heidelberg.de (S.J.D.); stefan.schoenberg@umm.de (S.O.S.); 3 Department of Surgery, University Medical Center Mannheim, Theodor-Kutzer-Ufer 1-3, D-68167 Mannheim, Germany; Michael.Keese@umm.de

**Keywords:** deep learning, computed X ray tomography, abdominal aortic aneurysm, image classification, interpretable artificial intelligence

## Abstract

Abdominal aortic aneurysms (AAA) may remain clinically silent until they enlarge and patients present with a potentially lethal rupture. This necessitates early detection and elective treatment. The goal of this study was to develop an easy-to-train algorithm which is capable of automated AAA screening in CT scans and can be applied to an intra-hospital environment. Three deep convolutional neural networks (ResNet, VGG-16 and AlexNet) were adapted for 3D classification and applied to a dataset consisting of 187 heterogenous CT scans. The 3D ResNet outperformed both other networks. Across the five folds of the first training dataset it achieved an accuracy of 0.856 and an area under the curve (AUC) of 0.926. Subsequently, the algorithms performance was verified on a second data set containing 106 scans, where it ran fully automated and resulted in an accuracy of 0.953 and an AUC of 0.971. A layer-wise relevance propagation (LRP) made the decision process interpretable and showed that the network correctly focused on the aortic lumen. In conclusion, the deep learning-based screening proved to be robust and showed high performance even on a heterogeneous multi-center data set. Integration into hospital workflow and its effect on aneurysm management would be an exciting topic of future research.

## 1. Introduction

Abdominal aortic aneurysm (AAA) is a potentially life-threatening condition [1,2,3]. Possible rupture is associated with high mortality exceeding 50% [4,5,6]. In clinical routine, a small AAA, as a low prevalence disease, may be identified as a co-finding on abdominal computed tomography (CT) images performed for various reasons. The focus on other clinical questions and the time-consuming nature of a detailed AAA analysis might lead to underreporting and delayed diagnosis [7]. Therefore, patients might become discharged without detection of an early AAA. This may potentially lead to a delay in treatment since surveillance programs have been shown to be of benefit [8]. Ultimately, spontaneous rupture can be prevented if larger AAAs are treated surgically or interventionally [9]. Deep learning enables fast and highly accurate analysis of image data, and therefore, seems highly suited to contribute to the management of AAA.

With deep learning being introduced for various use-cases in medicine, a new and promising era of technical support and guidance for physicians is emerging [10]. Research effort has recently been made towards utilizing deep learning for AAA detection, segmentation, and prognostic evaluation. Mohammadi et al. [11] and López-Linares et al. [12] both described a 2D convolutional neural network (CNN)-based cascading pipeline for automated detection and segmentation of AAA in abdominal CT scans. Habijan et al. [13] have applied a 3D U-Net with deep supervision to segment AAAs in CT. These advances are useful for intervention planning [2]. Algorithm-based AAA growth prediction from CTA scans [14,15,16] and its potential for detection of aortic dissection and monitoring of endovascular aneurysm repair (EVAR) therapy complication has been shown [17,18,19].

A robust and automated algorithm that can be included in routine clinical workflow remains a great challenge [20]. The lack of algorithm generalizability is a central obstacle, which usually is caused by development on highly preselected data sets containing scans from a limited number of scanners and mostly exclusively CTA contrast phases.

The aim of this study is to develop and validate an easily trainable and fully automated deep learning 3D AAA screening algorithm, which can run as a background process in the clinic workflow. The main requirements are robustness, reliability, and precision as well as automation, whereas the algorithm training and clinical application should be feasible with minimal effort.

## 2. Materials and Methods

### 2.1. Patients and Data Sets

Institutional Review Board (IRB) approval for this study was obtained from the ethics committee II of the Medical Faculty Mannheim, Heidelberg University (2016-863R-MA, 17 November 2016).

We acquired the training data set from our radiology information system (RIS) based on keyword queries (for details see Appendix A). The presence of an AAA was reported by two radiologists, of which one had at least five years of experience. The studies were reviewed by a radiology resident with 2.5 years of experience in the interpretation of vascular CT scans to ensure data quality and reproducibility. The abdominal aorta was considered aneurysmatic when the aorta exceeded a 50% focal increase of its diameter [21]. Special care was taken to ensure that the data set contains scans of AAA of various size and shape with multiple co-findings and various devices such as intra-arterial stents and metallic interferences being present to represent a realistic spectrum of clinical data.

**Figure 1:**
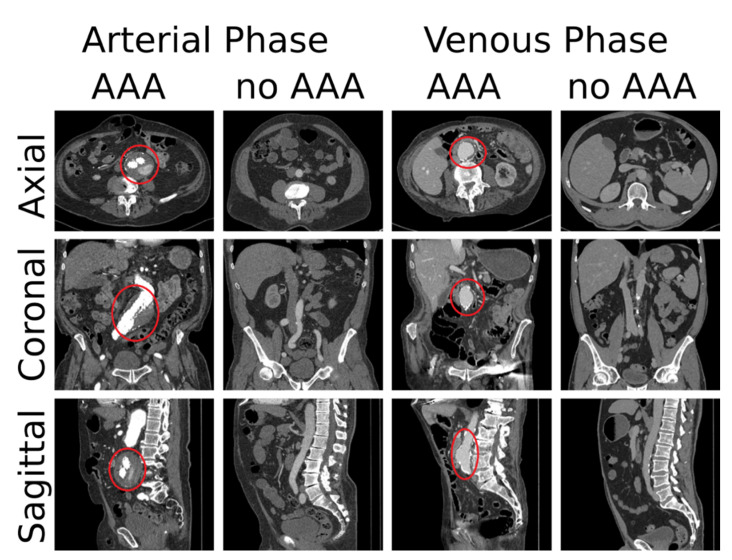
Four example cases from our training data set. For each case one slice of each principal anatomical plane is shown. The location of the AAAs is marked with a red circle.

Annotation of the training data set was performed in two ways. Firstly, each CT scan was assigned to one of two classes (0: no AAA, 1: AAA). Secondly, the axial position of the origin of the most cranial left renal artery for the aorta was noted as an anchor point in every scan, which was subsequently used to automatically extract a sub volume of standardized size during training. Four example cases are shown in Figure 1. Distribution of classes and further statistics of our data set are listed in Table 1.

For validation of the algorithm, we collected an additional verification data set from four publicly available sources (TCIA [22], IRCAD [23], CHAOS [24], BTCV [25]) and from our RIS as for the first data set. We excluded volumes from the public data, which do not cover the full abdomen, resulting in a total number of 50 cases (TCIA: 23, IRCAD: 9, CHAOS: 8, BTCV: 10). These scans originate from a broad spectrum of hardware of different manufacturers (Siemens Healthineers, Erlangen, Germany; Philips, Amsterdam, The Netherlands; Canon Medical Systems, Otawara, Japan). A total of 56 new cases were extracted from our PACS. Each CT scan was again assigned to one of the two classes (0: no AAA, 1: AAA). No further annotation was performed.

### 2.2. Networks, Preprocessing and Training

**Figure 2:**
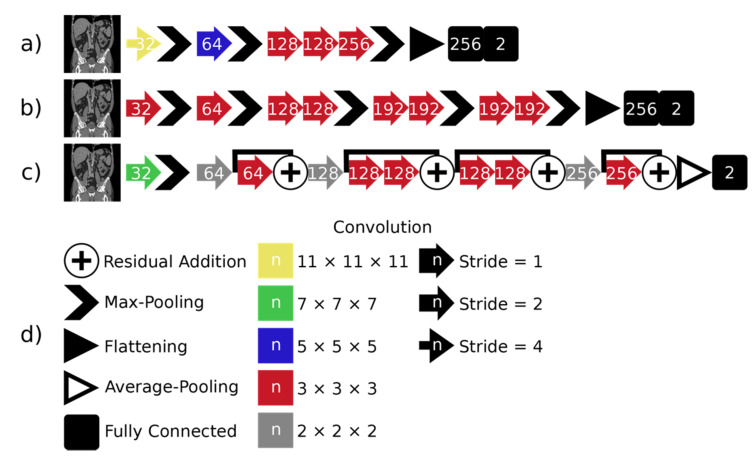
The three architectures used for classification are shown on the left: (**a**) 3D AlexNet, (**b**) 3D VGG and (**c**) 3D ResNet. All operations are given in the legend on the right of (**d**). The number of channels resulting from operations is marked by the number on the operation symbol.

We extended three established architectures from literature to be applied to 3D image classification: AlexNet [26], VGG-16 [27] and ResNet [28]. We altered the described architectures to economize the memory consumption. All networks use the ReLU activation function, except for the last layer, where softmax is used. The architectures are shown in Figure 2.

We use stratified 5-fold cross-validation on the initial training data set. The data are split into five disjoint test sets, each consisting of 36–39 cases. For each fold, one of these test sets is used and 6 cases are selected for validation from the non-test data. The remaining non-test cases are used for training. The 3D patches of 320 × 384 × 224 voxels are extracted from the CT volumes. This reduces memory requirements and removes the parts of the image depicting air and the patient table. For prediction, the patch is centered on the center of the anchor point slice. The densities of the CT images are windowed to range between [−200HU,400HU], thus covering an extended soft tissue range, and then mapped to the interval [−1,1]. All images are resampled to a spacing of 0.9 × 0.9 × 1.5 mm, which equals the median resolution of the data set.

During training, the anchor point slice is shifted by up to ten voxels along the cranio-caudal axis and the patch center is randomly positioned on the slice. Furthermore, data augmentation is also performed via rotation around the cranio-caudal axis with angle α ∈ [−12.6°,12.6°] to mimic possible patient positions and scaling by up to 10% to mimic possible patient sizes. Additionally, density jittering by up to ± 3 HU is applied. Details of the implementation and training of the networks are provided in Appendix B and Appendix C.

### 2.3. Layer-Wise Relevance Propagation

Layer-wise relevance propagation (LRP) allows the calculation of voxel-wise decomposition of the decision of a CNN. It can thus be used to provide interpretability for CNNs [29]. The relevance is propagated layer-wise from the network output back through the network, until the input layer is reached. The result equals a relevance map, which provides a relevance value on the output class for each single input value. In our maps, positive relevance values indicate a relevance for the AAA class, while negative values indicate a relevance against the AAA class. We normalize the maps by their sum to standardize them [30,31].

### 2.4. Fully Automatic Screening

To apply the screening algorithm fully automatically, an automatic abdominal image region extraction was developed. The underlying algorithm first analyzes the HU distribution along the z-axis in the HU value range of soft tissue to determine a lower and upper bound of the abdomen. The abdomen center is then determined based on the distribution of high HU values, corresponding to the area between the top of the hip bone and the lower ribs. A subvolume is then extracted based on the abdomen center and fed to the network for classification. Using this fully automatic screening, the 106 cases from the second data set were processed.

### 2.5. Evaluation

We employ five metrics to assess the quality of the predicted classification. We use a discrimination threshold (DT) of 0.5 for all four binary metrics. They are derived from four outcomes: true positive (TP) represents a sample correctly identified as AAA, true negative (TN) denotes a non-AAA sample correctly classified as such, false positive (FP) is an AAA sample falsely identified as a non-AAA case and false negative (FN) denotes a non-AAA sample being misclassified as AAA. From this, we calculate the accuracy (A), the precision (P), the true positive rate (TPR), the false positive rate (FPR) and the F1 score (F1) [32]. These measures compare the predictions of the networks to the labels. The receiver operating characteristic (ROC) curve plots the ratio between TP and FP decisions, when the discrimination threshold of a binary classifier is varied. It evaluates the performance based on the predicted probabilities of the networks and the labels. The area under the curve (AUC) provides a metric for the classifier performance.

To assess the correspondence of the relevance maps with the aorta we use a 5-point Likert scale, which is used to measure the agreement between the decision relevant region of the algorithm with the aortic location determined by a human radiologist [33]. Positive and negative relevance values were considered equally for the assessment. The influence of the aorta on the network decision is scored as: 1 (no relevance in the aorta), 2 (low relevance in the aorta), 3 (medium relevance in the aorta), 4 (high relevance in the aorta) and 5 (very high relevance in the aorta).

## 3. Results

To assess the performance of the CNN classification we performed *four* experiments. Firstly, we trained and tested three architectures. This was only performed for one-fold of the initial data set. Secondly, LRP is applied to the best network from Experiment 1 to validate that the network decision is based on the correct region of interest (ROI). Relevance maps were scored by a radiologist with 2.5 years of experience in vascular imaging. Thirdly, the training of the best network is repeated for the four remaining folds to verify repeatability. In the fourth step, fully automatic screening using the best network is tested on the second verification data set.

### 3.1. Results of the First Experiment (Network Comparison)

**Figure 3:**
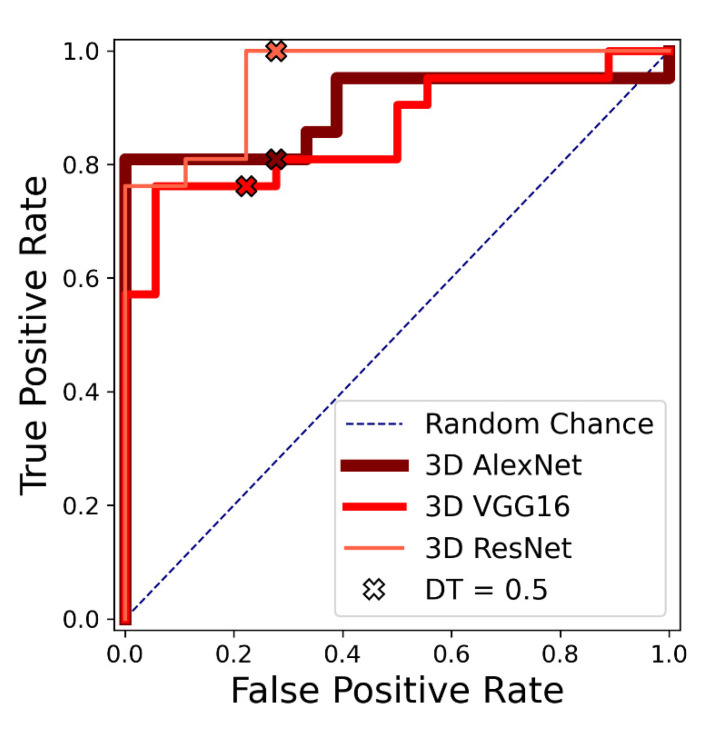
Experiment 1: Receiver operating characteristic (ROC) curves for 3D VGG, 3D AlexNet and 3D ResNet on one test fold of the first dataset. Performance with discrimination threshold (DT) = 0.5 is marked with an x for each network.

The results of the evaluation metrics for the first experiment are listed in Table 2. The ROC curves for the three networks are shown in Figure 3. The 3D ResNet achieved the best results according to all five metrics. Out of the 3D ResNet’s five false positive cases, two cases showed a pre-aneurysmatic enlargement.

### 3.2. Results of the Second Experiment (LRP Maps)

**Figure 4:**
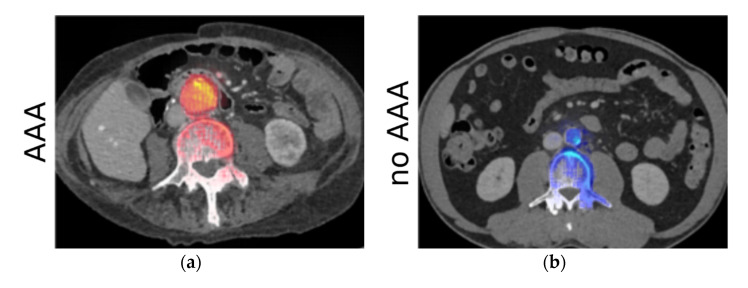

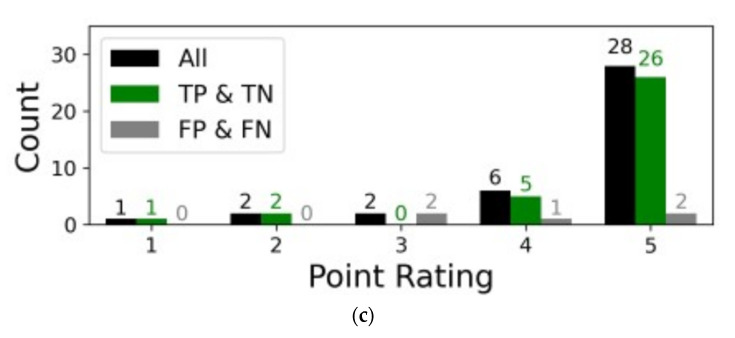
Experiment 2—results of the analysis using LRP. (**a**,**b**) show relevance maps for predicting the AAA super-imposed on the CT images for two example cases. (**a**) Shows a case with AAA, while (**b**) shows a case without AAA. Positive values are shown in red with high values in yellow, while negative values are shown in blue with high values as light blue. The high relevance around the aorta confirms that the networks correctly learned to make the classification decision based on the ROI. (**c**) Shows the score distribution of the assessment by an experienced radiologist.

In the second experiment, the rating of the relevance maps resulted in an average score of 4.56 for correctly classified cases. The exact distribution can be seen in Figure 4c. Relevance maps for a case with and a case without AAA are shown exemplarily in Figure 4a,b, respectively. For the AAA class, the highest relevance values were present on the inner lumen of the aneurysm. There was one case where no relevance was present in the aorta. This patient had an extracorporeal membrane oxygenation (ECMO) tube placed in the vena cava. The relevance analysis showed that the network focused on the inferior vena cava instead of the aorta.

### 3.3. Results of the Third Experiment (Repeated Training of the Best Network) and the Fourth Experiment (Verification on a Additional Dataset)

The results from the third experiment-the evaluation metrics for the five folds and across all data (aggregated from the five folds) are listed in Table 3. Its performance on the verification dataset (fourth experiment) is listed in Table 4. Visual inspection of the results of the automated region extraction confirmed that a suitable subvolume was extracted for all cases in the verification dataset. The extraction had an average computation time of 2 s.

## 4. Discussion

The findings of this study show that our 3D ResNet demonstrated a high performance and robustness for fully automated AAA detection in abdominal CT scans. Our proposed network achieves an AUC of 0.971 and an accuracy of 0.953 on the verification data set. Based on these results, the architecture seems to be suitable for clinical screening purposes.

Ultrasound-based AAA screening has been described to decrease AAA mortality and increase effectiveness of treatment and has partly been introduced into clinical practice [34,35]. Automated screening for AAA on abdominal CT scans that have been acquired for various other reasons would add another chance of early detection of AAA, possibly supporting radiologists and clinicians in reporting, monitoring and the treatment of AAA [7].

Time-consuming manual annotation remains a major bottleneck of the training and validation of newly developed algorithms on large clinical data sets [36,37]. In contrast, the screening algorithm presented here was designed to be trainable on minimally annotated data. Setting of the anchor point was automated for the fully automated screening on the verification data via a heuristic approach but could also be realized by anatomical landmark detection [38]. Such automatization provides the opportunity for future training and validation on much larger data sets. A critical factor for the successful implementation of artificial intelligence systems into hospital workflows besides factors such as integration into the local standard operating procedures and clinical systems is a high robustness and reliability to establish the necessary trust into AI systems [39]. The LRP analysis presented here is able to generate a graphical analysis of the decision relevant areas of the network in the images and therefore, could be used for validation. PACS export of these results might significantly increase its clinical acceptance. Detailed effects on patient treatment remain unanswered and represent an exciting field of further research.

Different groups have already described pipelines and tools for detection and classification of AAA in CT scans. A general shift of focus towards compatibility with routine clinical implementation can be observed, however, this process remains challenging [11,20,40,41,42]. Different levels of performance have been reported for AAA related image processing tasks. Mohammadi et al. reported a high accuracy of successfully classifying 2D patches to show the aorta (0.986) [11]. For the prediction of an aortic dissection and aortic rupture using a 2D CNN a TPR of 0.900 and 0.889 as well as an AUC of 0.979 and 0.990 were reported by Harris et al., respectively [17]. Our 3D ResNet achieves a similar TPR and a comparable AUC value to these works. In general, the task of classifying an entire 3D volume is more complex than classifying selected 2D patches and we targeted a different problem. To the best of our knowledge, and based on our extensive literature research, the application of 3D CNNs to CT AAA screening has not been presented previously. Solutions which apply 3D CNNs for whole CT binary classification of other pathologies in CT data report accuracies of 0.918 (lung cancer [43]) and 0.93 (COVID-19 [44]).

It is important to note that the results achieved with the algorithm proposed in this study were derived from training on a heterogeneous data set of 187 images, which includes different contrast phases, scanners, and artifacts. Mohammadi et al. trained and tested their algorithm on a data set of solely ten patients with two patients having an “obvious” AAA [11]. Whereas in one of our previous studies [45], metallic interferences have been shown to negatively impact the CNN performance, in this study the accuracy remained high even with presence of these artifacts, most likely due to the inclusion of these in the training data set and improvements in data augmentation during training. Moreover, our results show—though only using a small dataset—that a robust algorithm could be trained that achieved an even higher accuracy on a differently composed unseen second dataset. Additional validation on a mixed dataset of external and internal abdominal CT scans on the one hand introduces the risk of verification on a dataset which does not entirely reflect clinical reality. The external data contained fewer metal artifacts and the AAA cases were often more simplistic cases. On the other hand, inclusion of these additional cases also increases CT data variability originating from international cohorts and different manufacturer hardware. It contributes to the understanding of the potential algorithm performance under different clinical conditions and the generalizability of the proposed method. The algorithm described here proved to be robust enough to achieve reliable results on the verification data set consisting of heterogeneous data from multiple sources.

The 3D ResNet, however, produced some FP results on the training data set. In two cases, early enlargements which were not aneurysmatic yet were detected. In another case, for one patient with an ECMO tube placed in the vena cava, the LRP revealed that the network focused on the inferior vena cava instead of the aorta. The classification result for this case was, however, correct. We assume that the ECMO tube was interpreted as an aortic stent by the network and therefore, caused the mislocated area of interest. On the verification data set, two FN cases were overserved. These cases showed very atypical shapes of the aneurysm where the aneurysmatic part was entirely thrombotic and the free-flown part had the shape of a regular aortic vessel. Further training on more data would probably solve this drawback. The aneurysm size and shape definition utilized for annotation seem critical for algorithm sensitivity, especially in small aneurysm.

### 4.1. Limitations

A limitation of our approach on the one hand is caused by the design of the algorithm as a binary classification task instead of object detection or segmentation. Screening could potentially also be solved with an object detection network, this would, however, require far more extensive annotations and is not guaranteed to provide a better performance. Our solution does not allow for automated segmentation and volumetry (which has already been achieved by other authors). While the very heterogenous datasets promote generalizability, the high differences in the slice thickness could also introduce a bias. However, in our approach, we interpolated the slice thickness as well as the in-plane resolution to the median resolution of the training data overcoming such problems. Future directions lie in the practical use of the technology developed within this study to notify clinicians in real-time. In addition, in cases of relevant AAA, a second pipeline could be triggered which includes fully automated AAA segmentation and analysis, similar to the one introduced by Lareyre et al. [41]. On the other hand, our study used a comparatively small patient cohort of 187 CT scans as a training data set, potentially limiting the dataset variability and thus, generalizability. However, the training was performed on a heterogeneous dataset derived from a large University Medical Center with a high variety of different findings. In addition, data augmentation methods were employed to simulate variations. 

In summary, the training and additional validation on unfiltered clinical data sets mark a step forward towards clinical implementation. Further testing on a much larger international cohort seems paramount for integration into the workflow of different medical sites that might differ in various factors. Training of the developed methodology on a larger cohort would be possible with reasonable extra effort and therefore, should be performed to ensure a broad generalizability.

From a medical and clinical perspective, it will be exciting to investigate if earlier detection of small AAA contributes to a more effective patient treatment and ultimately improvement of prognosis. A possible prospective observer study monitoring the effects of the technology on patient care would be a logical and necessary next step. Besides the algorithm performance, the way of its clinical introduction represents a highly important factor influencing the clinical success and as a major remaining question should be defined more clearly in upcoming scientific works.

### 4.2. Conclusions

This study demonstrated the feasibility of CNN-based fully automated detection of AAA in an unselected clinical data set of CT images. The generation of relevance maps contributes to the explainability of the decision process. Integration of this deep learning screening for AAA into the routine workflow might lead to improved patient monitoring, earlier diagnosis, and improved patient treatment with possible reduction of rupture risk.

## Figures and Tables

**Table 1:** Data set statistics.

	Training Set	Verification Set				
		TCIA	IRCAD	CHAOS	BTCV	Internal
Cases (total)	187	23	9	8	10	56
Cases in arterial phase	85	0	0	0	0	31
Cases in venous phase	102	23	9	8	10	25
Cases with AAA	100	0	1	0	1	56
Voxel resolution X/Y	0.9 ± 0.1 mm	0.8 ± 0.1 mm	0.7 ± 0.1 mm	0.7 ± 0.0 mm	0.7 ± 0.1 mm	0.8 ± 0.1 mm
Voxel resolution Z	1.5 ± 0.5 mm	1.0 ± 0.0 mm	1.6 ± 0.8 mm	1 ± 0.4 mm	3 ± 0.6 mm	1.5 ± 0.6 mm
Slices [min, max]	[101, 2687]	[187, 310]	[79, 260]	[95, 266]	[42, 148]	[101, 1323]

Data set statistics—resolution is given as median ± standard deviation.

**Table 2:** Comparison of three different networks.

Network	A	P	TPR	*F*_1_	AUC
3D AlexNet	0.769	0.773	0.810	0.791	0.899
3D VGG	0.769	0.800	0.762	0.780	0.860
3D ResNet	**0.872**	**0.808**	**1.000**	**0.894**	**0.931**

Experiment 1: Quantitative comparison of the classification quality of three different networks for one-fold. The best result for each metric is marked bold. A = accuracy; P = precision; TPR = true positive rate; F_1_ = F1 score; AUC = area under the curve.

**Table 3:** Results for 3D ResNet.

Fold	A	P	TPR	F_1_	AUC
1	0.872	0.808	1.000	0.894	0.931
2	0.821	0.850	0.810	0.829	0.929
3	0.838	0.850	0.850	0.850	0.919
4	0.944	0.947	0.947	0.947	0.961
5	0.806	0.773	0.895	0.829	0.885
All	0.856	0.841	0.900	0.870	0.926

Experiment 3: quantitative comparison of the classification performance of 3D ResNet across 5 folds. Last line shows the results computed across all test folds. A = accuracy; P = precision; TPR = true positive rate; F_1_ = F1 score; AUC = area under the curve.

**Table 4:** Performance on verification data set.

Fold	A	P	TPR	F_1_	AUC
All	0.953	0.949	0.966	0.957	0.971

Experiment 4: quantitative analysis of the classification performance of 3D ResNet on the verification data set.

**Figure A1:**
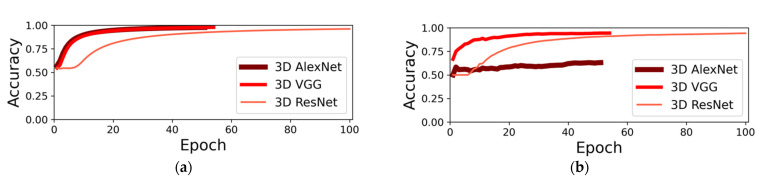
Average training (**a**) and validation (**b**) accuracy for all three networks across the training epochs. Each training is plotted up to the selected best epoch. The selected 3D ResNet trained for 100 epochs, while 3D VGG and 3D AlexNet trained 54 and 51 epochs, respectively. The 3D VGG and 3D ResNet both reached a high validation accuracy at convergence.

## Data Availability

The sources of the publicly available datasets used for composition of the verification dataset are listed in the references [22,23,24,25]. The data retrieved from our own clinical systems used for training and partly verification presented in this study is not publicly available due to patient privacy concerns, publication would not be covered by IRB statement.

## Abbreviations and Acronyms

AAA	Abdominal aortic aneurysm
AUC	Area under the curve
CNN	Convolutional neural network
CT	Computed tomography
CTA	Computed tomography angiography
DT	Discrimination threshold
ECMO	Extracorporeal membrane oxygenation
EVAR	Endovascular aneurysm repair
LRP	Layer-wise relevance propagation
PACS	Picture archiving and communication system
ROC	Receiver operating characteristic
ROI	Region of interest
RIS	Radiology information system

## Appendix A. Data Aggregation and Dataset

For the data aggregation of the training dataset, keywords used to find suitable cases in the PACS were (translated): ”aortic aneurysm”, ”aneurysm”, ”abdominal aneurysm”, ”AAA”, ”no aneurysm”, ”no AAA” within the radiology report. This search strategy yielded a substantial number of reports confirming the presence of AAA and other reports denying presence of AAA. The scans for algorithm training were extracted from our picture archiving and communication system (PACS). The scans were derived from a total of seven different CT scanners (SOMATOM Force, SOMATOM Definition Flash, SOMATOM Definition AS 128, SOMATOM Sensation 64, SOMATOM Emotion 16, SOMATOM Emotion 16, Biograph mCT, all Siemens Healthineers, Erlangen, Germany) which were located at two different medical sites (being part of our department). Only contrast enhanced CT scans were included. Some scans were acquired 70 s post-contrast media application (venous), some in CTA contrast phase (arterial) and some in a mixed phase of 40–50 s post-application, which were classified as venous studies within this analysis.

## Appendix B. Networks

Background information about the networks utilized: AlexNet was proposed by Krizhevsky et al. for image classification [26]. Its first five layers are convolutional layers with three max-pooling layers located in between. The final three layers are fully connected layers. In this work, we modified the network to make it suitable for 3D classification by reducing the number of filters and employing only two fully connected layers.

The VGG-16 architecture was introduced by Simonyan et al. [27]. In contrast to the AlexNet it uses multiple 3 × 3 convolutions replacing large kernel-sized filters. Our 3D implementation has a lower number of filters and layers. Additionally, the first convolutional layer uses a stride of two in every spatial direction.

He et al. developed a CNN with residual connections called ResNet [28]. The residual connections are formed by adding feature maps from the beginning of a block of convolutions to the final feature maps. Other than these connections, the ResNet also uses an average pooling instead of flattening the feature values before the first fully connected layer. The ResNet only uses max-pooling for downsizing in the first layer, subsequently downsizing is performed via strided convolutions. It also employs a batch-normalization [46]. Similarly, to the other networks, we adapted the number of channels and layers. We also increased the stride in the first layer to two.

The training was performed using the Adam optimizer with a learning rate of 10^−3^. We used the binary cross entropy loss and apply a L2 regularization on the network weights. The regularization term is weighted by 10^−5^. We applied an early stopping to the training process: after the initial 20 epochs, we tested for the convergence criterion at the end of each epoch. The criterion is defined as the change of validation accuracy over the last twenty epochs falling below 10^−4^. For the application we then selected the weights with the highest accuracy on the validation data from the last 20 epochs. The convergence of the networks is shown in Figure A1. The 3D ResNet converged after the highest number of epochs. The 3D AlexNet showed a low generalization capability during the training. The training time for each network was 34 to 57 h. The average prediction time was 3 s.

## Appendix C. Implementation

The described networks were implemented using TensorFlow 1.12 [47] and Python 3.6. For the evaluation and the image processing we used SimpleITK 1.2.4, which provides a simplified interface to the Insight Toolkit (ITK) [48]. LRP was realized using the iNNvestigate Neural Networks! Toolbox 1.0.9 [49]. Training and testing were performed on a Windows Server 2016 with an Intel Core i7-7700K CPU, 64GB of RAM and a NVIDIA Quadro P5000 graphics card with 16 GB VRAM.
